# Building the Clinical Bridge: An Australian Success

**DOI:** 10.1155/2012/579072

**Published:** 2012-04-23

**Authors:** Marianne Wallis, Wendy Chaboyer

**Affiliations:** ^1^NHMRC Centre for Research Excellence in Nursing (NCREN), Griffith University, Gold Coast, QLD 4222, Australia; ^2^School of Nursing and Midwifery, Griffith University, Gold Coast, QLD 4222, Australia

## Abstract

Nursing effectiveness science includes primary, secondary, and translational, clinically focused research activities which aim to improve patient or client outcomes. It is imperative, for the successful conduct of a program of nursing effectiveness science, that a clinical bridge is established between academic and healthcare service facilities. An Australian example of the development of a robust clinical bridge through the use of jointly funded positions at the professorial level is outlined. In addition, an analysis of the practical application of Lewin's model of change management and the contribution of both servant and transformational leadership styles to the bridge building process is provided.

## 1. Introduction

In Australia, a relatively popular strategy, aimed at improving collaboration between clinicians and research focused academics, has been the appointment of a Professor of Nursing and/or Midwifery into a Clinical Chair position, jointly funded by a university and a health service or hospital (a joint Clinical Chair). There has been much commentary, especially in Australia, about the development of such positions [1–4]. What is not so clear from this commentary is what elements surrounding this strategy lead to successful collaborative partnerships and the development of a clinical bridge to enable nursing effectiveness science.

By nursing effectiveness science we mean primary, secondary, and translational, clinically focused research activities which aim to improve patient or client outcomes. Activities involved in nursing effectiveness science include systematic reviews and meta-analyses of previously conducted research studies, exploratory, correlational, and experimental clinical research studies, and translational research studies aimed at changing clinical practice. In this paper we outline the antecedents that shaped one university-health service collaboration and the change management strategies which helped construct the clinical bridge. We also explore how different types of leadership facilitated the achievement of outcomes, and finally we comment on future directions for nursing effectiveness science.

## 2. The Context

Joint Clinical Chair positions in nursing emerged, in Australia, in the late 1980s. By 1996 there were 20 joint Clinical Chairs in Australia [5] and currently there are many more. Generally, these positions are appointed at the level of Professor although some have been appointed at the level of Associate Professor. There is also a distinction between “generic” joint Clinical Chairs (i.e., Professor of Nursing, Professor of Midwifery or Professor of Clinical Nursing Research) and “specialist” joint Clinical Chairs (i.e., Professor of Critical Care Nursing). In the early days, the focus, goals, and structures associated with these positions were largely driven by individuals. At a two-day symposium, in Sydney, in the late 1990s, a number of incumbents and stakeholders described the positions that were then in place and it was clear that there was no coherent model for what the joint Clinical Chairs would do, whether the focus should be broad or narrow and how they would work across academic and service organisations (personal communication, M. Wallis 2011). Qualitative research into the perspectives of a number of the early incumbents of these positions confirmed that there was a “diversity of arrangements between university and health sector partners in establishing their respective roles” [2, page 165].

The main challenge that was identified early in the development of these positions was the potential for unrealistic workloads [1]. As Darbyshire [4] expressed it.

*Not only will the professor be expected to be a top researcher, winning grants, establishing research programmes, publishing, supervising, teaching, leading, consulting, examining, networking, presenting and more, but in the service sector they will also be expected to be a kind of super staff development guru and contract researcher whose role is no less than to change the nursing culture of the organization, improve care and service provision quality and give the “research answers” that will extinguish the most troublesome clinical, professional or organizational fires of the day. “After all”, you can hear the hospital executive thinking, “we pay half of their salary, so we may as well use them.” (p. 2595).*

This quote also alludes to the fact that universities and health service providers may not only have quite disparate cultures, they may also have dissimilar goals and expected outcomes. Despite these problems, there continues to be university and healthcare organisational commitment to these positions. In 2000, the Faculty of Nursing, at Griffith University, in southeast Queensland, Australia, and the Division of Nursing in one of the local healthcare services, Gold Coast Health Service District, appointed a joint Chair, Clinical Nursing Research.

## 3. The Beginning of the Clinical Bridge

If a clinical bridge is to be built to support nursing effectiveness science, the first things that have to be in place are senior people with vision and the resources to support that vision [3, 4, 6]. On the Gold Coast, in Queensland, Australia, in 1997, the two people with vision were the Dean of the Faculty of Nursing, at Griffith University, and the Executive Director of Nursing (EDON) of the local health service. These two transformational leaders [7] had a mutual respect for the contribution of healthcare services and academic institutions to professional nursing and to client outcomes. While the role of the EDON necessarily focused on service delivery, there was a clear understanding of the value of a collaborative approach to healthcare that incorporated research and education. The Dean was clear about the critical importance of clinical practice to the discipline and the need for clinically informed and clinically relevant research. Together, because of their perspectives, they developed a plan to address the theory-practice gap that was evident in Australia at the time [8].

The plan that was developed, and eventually endorsed by senior management in both organisations, was to appoint a joint Clinical Chair. The Dean and EDON had carefully considered what was required within the local context and had decided that this needed to be a transitional position. The joint Clinical Chair was not designed to last forever and the need for change and evolution of the role were incorporated from the beginning. It would have a generic focus (i.e., nursing) but would specifically include in the title the word “research” to signal to all parties the main focus of the position. A contract was drawn up for a joint Chair, Clinical Nursing Research, and appropriate resources were allocated not just to the funding of the position, but also to research nurse support at the clinical site. The incumbent was to be employed by the University, but half the salary was paid by the health service. Office space was provided within both organisations although more time was to be spent on site at the hospital.

Based on their assessment of the clinical context, the Dean and EDON decided that the first step was to appoint a leader who could drive the development of the people, the infrastructure and the capacity required to build an ongoing program of clinical nursing research. This position was always seen by all parties as the beginning of a collaboration that would be focused on building a clinical research culture within the health service and on developing then the Faculty of Nursing (now School of Nursing and Midwifery) into a leading nursing research facility. As such, the second vitally important element to the successful development of the joint Clinical Chair was clarity and unity related to the anticipated outcomes for the position [3]. The Dean and the EDON were both broadly in agreement that the goals for the position were to increase research funding from external, competitive funding bodies, increase peer-reviewed publications, and increase the number of Ph.D. completions; and they allowed the first incumbent to negotiate the key performance indicators and the timelines.

## 4. Build It and They Will Come

**Figure 1:**
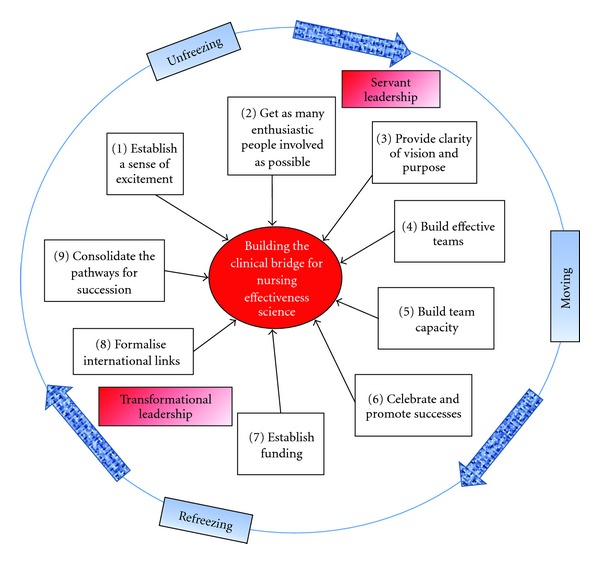
The cycle of organisational change management involved in building a clinical bridge for nursing effectiveness science.

When building the clinical bridge to facilitate effectiveness science on the Gold Coast, an approach was taken that integrated Lewin's [9] model of unfreezing, moving, and refreezing with leadership approaches that included both servant leadership [10] and transformational leadership [11].  Figure 1 depicts the cyclical nature of the bridge building process, outlines some of the key elements, and indicates leadership approaches that can bring success.

In 2000, when the joint Chair, Clinical Nursing Research, was appointed, a sense of urgency and excitement was palpable within both organisations. As Kotter [12] suggests establishing this sense of excitement is important as it motivates people to get outside their comfort zones and contributes to Lewin's unfreezing phase. Local and regional promotion of the new position built on this excitement and an initial process of meeting and discussing potential collaborative endeavours provided the opportunity for the incumbent to commence the position with a servant leader focus. Servant leadership is as much about followership as it is about leadership. Servant leaders begin by discovering the needs of the people they serve [10]. They give priority to their relationships with followers rather than their relationship with the organization and emphasise followers' holistic needs, development, and autonomy [13]. Servant leaders exercise their influence through the transformation of their followers. In contrast, transformational leaders focus on mobilizing followers to achieve “performance beyond expectations,” which is the ultimate priority of the organisation [7].

If any leadership position is to function effectively, there also have to be willing followers and collaborators [14]. Both organisations had enthusiastic academics and clinicians who had already begun to establish links around specialist clinical education and clinical research projects. One of the things that made the bridge easier was the movement of specialty postgraduate nursing education from the hospital setting to the university which occurred a few years prior to the establishment of the joint Clinical Chair. As another element in the unfreezing of the clinical and academic environments (see Figure 1), specialist Master-of-Nursing programs (e.g., Critical Care Nursing, Emergency Nursing, etc.) were offered in a university-hospital partnership model. Consequently, university academics and hospital-based clinical educators had established excellent working relationships. Also because the courses were at the Master-of-Nursing level, the students (many of whom were clinical leaders) received some research training, increasing their appreciation of the value of clinical research. In addition, one Ph.D. prepared academic was already working with a clinical manager, a clinical specialist, and an educator and building a strong track record of research around ICU nursing.

Despite this ground work, in 2000 the Gold Coast Health Service District was not a research-ready, let alone a research intensive, healthcare facility; it was primarily focused on service provision for members of the local community. The Faculty of Nursing at Griffith University was similarly just beginning its development as a centre for clinical research. There were many researchers beginning their careers, but there was little focus and no critical mass of researchers who were working together either in the University or the Health District. As southeast Queensland became the focus of internal migration within Australia, and the population along the corridor from Gold Coast to Brisbane boomed, it became clear that there would need to be a huge expansion in healthcare services and that this presented a unique opportunity to build a research centre that would not only improve the health of Queenslanders, but also could develop into a centre of international repute.

As discussed by Ba Banutu-Gomez [15, page 147] “the central role of a servant leader is to establish a sustainable strategic vision for the organisation ….” Thus at the end of 2002, as a strategy both to complete the unfreezing stage and then to direct the moving stage of change (see Figure 1), the joint Clinical Chair and other leaders in the nursing faculty, building on their beginning successes in collaborative clinical research, wrote a proposal for a research centre to focus on clinical innovation. The Griffith University Research Centre for Clinical and Community Practice Innovation (RCCCPI) was launched in 2003. While initially RCCCPI was a collaboration between the Griffith University School of Nursing and Midwifery and Gold Coast Health, it expanded quickly and membership now includes researchers in six major teaching hospitals in southeast Queensland and has strong links to universities in Australia, Canada, the UK, and the USA. There were a number of iterations of the programs within the Centre until eventually significant multidisciplinary teams of people coalesced around the areas of acute and critical care; ageing and older people; nutrition; and maternity and family. The leadership of RCCCPI maintained and grew the strong links with the joint Clinical Chair and the growing number of clinicians engaged in research in the health service. More clinicians enrolled in and graduated with research degrees and the joint Clinical Chair and senior research colleagues started to acquire competitive grants.

In order to maintain the momentum and to keep the change process moving, a number of strategies were employed that were designed to build effective teams and to build team capacity (see Figure 1). One of these strategies was to conduct workshops for clinicians related to evidence-based practice. University-based academics as well as the joint Clinical Chair and hospital-based researchers all contributed to these workshops. Funding was sought to allow nurses to attend these workshops in work hours, and an evaluative research study of these workshops clearly indicated positive attitudinal change in clinicians [16]. The health service then had a cohort of senior clinicians who were primed with a critical approach to their practice and ready to “move” into research. Having a joint Research Seminar Series between RCCCPI and the health service meant that research conversations between academic and clinical nurses continued that movement. Other early strategies that worked included setting up a Visiting Nursing and Midwifery Research Fellow Program and setting up a mentorship program for clinicians involved in research. The Visiting Nursing and Midwifery Research Fellows were Griffith University nurse researchers who would come and work in an honorary capacity with clinicians in particular specialist areas. They were appointed in areas such as ICU, community care, mental health nursing, and midwifery and they joined teams of clinical nurses and midwives to help develop programs of research in specialist areas. When in the hospital setting, they were supervised by the joint Clinical Chair.

Funding for research activities, in the early days, was a challenge, as is commonly the case [4]. Establishing a funding stream for research activities is, however, vital if the clinical bridge is to be sustained and the change to a research intensive clinical culture is to be “refrozen.” So various strategies were employed including: always having research teams that incorporated academics and clinicians (something that appeals strongly to the organisations that fund clinical research); focusing research activity on areas of high interest to funding bodies; approaching local health service power brokers with clearly articulated proposals for projects of mutual benefit; accepting appointments to strategic committees, at higher levels of government; linking with medical colleagues to establish research positions within the clinical environment; and finally making sure that there were working relationships and strategic alliances at all levels in the health department. Together all these strategies, and the work of a growing number of collaborative groups, began to bear fruit.

## 5. Growing Success

As a way of both measuring and celebrating the successes of the first few years, the health service produced a Compendium of Nursing Research every second year. Both the health service and the university produced many reports about their total activities, but the Research Compendium served to celebrate, specifically, the successes of teams that involved the clinical nursing staff and served to highlight the strong connections between the two organisations.  Table 1 is a reproduction of the table originally published in the 2009 Compendium.

In the early phase of building the clinical bridge, the successes steadily accumulated but it was not until 2005 that significant funding successes began to flow through to research outputs such as publications in international journals. This initial period saw the success of a servant leader approach. The joint Clinical Chair and Visiting Nursing and Midwifery Research Fellows worked closely with clinicians and followed their suggestions for areas of research focus. By weaving this approach with a transformational leadership approach, which involves shifting the values, beliefs, and needs of followers to meet organisational goals through empowering and building capacity in the workforce [17], a firm foundation for building a program of nursing effectiveness science was established (see Figure 1).

In the initial period, while a successful track record was being established, by the joint Clinical Chair and teams of clinicians, there were two interlinked strategies that helped the health service appreciate the value of the joint Clinical Chair. These strategies were increasing the number of clinicians who successfully completed research-based Masters Programs and engagement in smaller projects that resulted in clinical practice changes within the local health service. Examples of research studies that resulted in changes in practice are listed in Table 2.

By 2005 RCCCPI was expanding and the number of university researchers working in collaboration with clinicians, in a number of health services in southeast Queensland, was expanding. These nurse researchers brought additional skills in effectiveness science that, in turn, led to greater grant success. Randomised controlled trials and translational research studies became the norm for the group. One team was successful in attracting over AUD 500,000 for a randomised controlled trial of routine removal of peripheral intravenous catheters compared to removal on clinical indication, from the Australian National Health and Medical Research Council (NHMRC) (similar to but much smaller than the US National Institutes for Health). The NHMRC traditionally allocates less than 3% of its funds to research lead by nurses, so this was a considerable breakthrough for the team. This grant success, and the ultimately successful completion of the research project, would not have been possible without the excellent collaborative working relationships, developed over these past years, between the university academics and their clinical partners, in a number of hospitals in southeast Queensland.

## 6. The Next Step

Under the transformational leadership of the RCCCPI Director, researchers were able to formalise links with a number of international research teams, continue to strengthen their clinical collaborations, and expand their research capacity. Health-service-based clinical nurse researchers were offered adjunct positions with RCCCPI (i.e., these researchers were employed by their hospitals but were given adjunct or honorary positions in Griffith University). One of the RCCCPI adjunct Professors at another hospital in southeast Queensland had a strong link with the Cochrane Collaboration Wounds Group. This connection allowed the group to develop skills in undertaking Cochrane systematic reviews. These activities were successfully refreezing the academic and clinical environments into one where research was the norm, especially in the acute care, in-patient areas of a number of southeast Queensland health services (see Figure 1). The RCCCPI Director with assistance from other Griffith University Professors and the joint Clinical Chair then led the team in a successful application to become an NHMRC Centre of Research Excellence. The NHMRC manages the Australian government's competitive grant process that funds health-related research. In addition to managing the competitive process for Project Grants, Program Grants, and a variety of Research Fellowships, it also funds a very small number of centres for research excellence. The Centre for Research Excellence in Nursing Interventions for Hospitalised Patients (NCREN), established in 2011, focuses on providing evidence to improve the nursing care of a broad range of hospitalised patients who have compromised skin integrity and/or require symptom management. These two particular areas were chosen because of (1) their high risk and high cost; (2) the research expertise within the team; and (3) Registered Nurses are largely responsible for patient care related to both skin integrity and symptom management. This is the first ever NHMRC Centre of Research Excellence to be awarded to a centre focused on nursing. NCREN now sits within RCCCPI as one strand of research. Both university academics (including the joint Clinical Chair) and clinicians in the local health service are named investigators within this new centre for research excellence, a clear statement of the strength of the clinical bridge.

In line with the thoughts of the original leaders who envisioned the joint Chair, Clinical Nursing Research, it became clear that the success of the collaboration and the strength of the clinical bridge could support the growth and evolution of the joint Clinical Chair position. In 2006 Griffith University appointed a specialty Clinical Chair with another southeast Queensland hospital in Critical Care Nursing. Then in 2011 Griffith University and the health service on the Gold Coast were successful in securing funding for a Chair in Midwifery from the Department of Health. When the incumbent of the Chair, Clinical Nursing Research, resigned in early 2011, the health service and Griffith University decided that the groundwork had been accomplished and that it was time for the Chair to evolve from a generic Chair to a more focused Chair in Acute and Complex Care Nursing. There are also longer term plans to appoint clinical chairs in other specialist areas such as mental health nursing, aged care nursing, and community care.

NCREN has been funded for five years and the team of researchers involved are back at the first element in the model depicted in Figure 1. The increased research activity demanded by NCREN's success will put stress on the clinical bridge. As clinicians struggle to maintain their standards in the face of increased clinical demand and now increased research demand, it behooves the research leadership to remain cognisant of the need for followership which is inherent within the models of servant and transformational leadership which have been manifestly successful to date.

## Figures and Tables

**Table 1:** GCHSD nursing research outputs 1999–2008.

Biennium	Total funding	Conference posters	Conference papers	Peer-reviewed publications
1999-2000	$700	8	3	2
2001-2002	$220,000	1	21	15
2003-2004	$270,000	5	20	24
2005-2006	$643,000	1	27	43
2007-2008	$1,187,255	5	48	45

**Table 2:** Changes in clinical practice following local primary, secondary, and translational research.

Clinical area involved	Change in practice
Community services	(i) Implementation and funding of the Waterworx Continence Centre. This community-based model of service provision was taken up by other Queensland health districts.

Intensive care unit	(i) Implementing and evaluating the introduction of an ICU discharge liaison nurse position.
	(ii) Production of patient/family information booklets for ICU.

Coronary care unit	(i) Implementation and evaluation of nurse-led care for heart failure patients.

General medical-surgical areas	(i) Management of peripheral IV infusions in children and in adults: removal on clinical indication.
	(ii) Followup of patients following total hip replacement surgery.
	(iii) Improved consent procedures and appropriateness of decision making for blood transfusions.
	(iv) Clinical trial of different pin-site management protocols.
	(v) Review of predischarge patient information in surgical wards.
	(vi) Production of evidence-based clinical guidelines and patient information on the management of constipation in middle-aged adults. These materials are now produced by the Australian Department of Health and Ageing.
	(vii) Development and implementation of the Renal Unit Clinical Nutrition Decision Support Algorithm.

Mental health	(i) Introduction and evaluation of patient-focused care in an acute psychiatric setting.
	(ii) Introduction and evaluation of a social development programme for young men with schizophrenia.

Aged care	(i) Design and evaluation of a dementia training program for aged care workers.

Paediatrics	(i) Introduction and evaluation of the program for adolescents with chronic illness.
	(ii) Introduction and evaluation of the Fun Not Fuss with Food program, ongoing involvement with the project suggests that this will be implemented statewide.
